# Regulation of macrophage-mediated chronic inflammation by JAK inhibitors

**DOI:** 10.1186/ar3679

**Published:** 2012-02-09

**Authors:** Anna Yarilina, Kai Xu, Chunhin Chan, Lionel B Ivashkiv

**Affiliations:** 1 Arthritis and Tissue Degeneration Program, Hospital for Special Surgery, USA; 2 Weill Medical College of Cornell University, New York, NY, 10021 USA

## 

Small molecule inhibitors of the Janus kinases (JAK) have been developed as anti-inflammatory and immunosuppressive agents and are currently subjects of clinical trials. Tofacitinib/CP-690,550 (more potent in inhibiting JAK3 and JAK1) and Ruxolitinib/INCB-018424 (selective inhibitor of JAK1/2) have demonstrated clinical efficacy in rheumatoid arthritis (RA), however, the exact mechanisms that mediate the inhibitory effects of these compounds are not known.

In this study, we examined the effects of CP-690,550 (CP) and INCB-018424 (INCB) on inflammatory responses in human macrophages (hMΦs). In our study, we used long term exposure to TNF as a model of chronic inflammation to investigate mechanisms regulating hMΦ activation and functions, and have shown that TNF can activate an IFN-JAK-STAT-dependent autocrine loop that regulates expression of pro-inflammatory chemokines and interferon stimulated genes (ISGs), followed by an increase of NFATc1, that regulates osteoclastogenesis.

As expected, both inhibitors abrogated TNF-induced STAT1 activation and expression of genes encoding inflammatory chemokines (*CXCL9, 10, 11 *and *CCL5*) and ISGs (*IFIT1 *and *2*, *IRF7*). Interestingly, both compounds attenuated a late wave of *IL-1 *induction and nuclear expression of NF-κB subunits. Furthermore, ex vivo treatment with inhibitors decreased *IL-1 *and *IL-6 *expression in synovial MΦs isolated from the patients with arthritis. Next, we analyzed the effects of JAK inhibitors on TNF-induced osteoclastogenesis and discovered that both compounds augmented nuclear levels of NFATc1 and cJun, followed by increased formation of TRAP positive multinuclear cells. Lastly, we examined an in vivo effect of CP on innate immune response in arthritis using K/BxN serum transfer arthritis model and found that CP treatment significantly inhibited inflammation and joint swelling. Taken together, our data suggest that JAK inhibitors can affect inflammatory responses in hMΦs and thus, can target both acquired and innate immunity in RA and other chronic inflammatory diseases.

